# Pulmonary renal syndrome in a patient with vasculitis: Case report and review of literature

**DOI:** 10.12669/pjms.316.8391

**Published:** 2015

**Authors:** Aijaz Zeeshan Khan Chachar, Omer Sabir, Irfan Haider, Imrana Tanvir, Kashif Rafique, Nauman Tarif

**Affiliations:** 1 Aijaz Zeeshan Khan Chachar, MBBS. Department of Medicine, Fatima Memorial Hospital, University of Health Sciences, Lahore, Pakistan; 2 Omer Sabir, FCPS. Department of Medicine, Division of Nephrology, Fatima Memorial Hospital, University of Health Sciences, Lahore, Pakistan; 3 Irfan Haider, FCPS. Department of Medicine, Fatima Memorial Hospital, University of Health Sciences, Lahore, Pakistan; 4 Imrana Tanvir, FCPS. Department of Pathology, Fatima Memorial Hospital, University of Health Sciences, Lahore, Pakistan; 5 Kashif Rafique, MBBS. Division of Nephrology, Fatima Memorial Hospital, University of Health Sciences, Lahore, Pakistan; 6 Prof. Nauman Tarif, DABIM, DABIN. Division of Nephrology, Fatima Memorial Hospital, University of Health Sciences, Lahore, Pakistan

**Keywords:** Cyclophoshamide, Granulomatosis with polyangiitis, Plasmapheresis, Small vessel vasculitis

## Abstract

Granulomatosis with polyangiitis (GPA) previously known as Wegner’s granulomatosis, is a small vessel vasculitis that preferentially involves capillaries, arterioles and venules, presenting as multisystemic disease classically with alveolar haemorrhage and renal insufficiency. We report a case of GPA diagnosed on history, clinical findings and supported by imaging and very high levels of C-ANCA. Renal biopsy confirmed the typical histopathological findings. We discuss herein the management of the case and review of literature.

## INTRODUCTION

Granulomatosis with polyangiitis (GPA), previously known as Wegner’s granulomatosis, is a small vessel vasculitis due to antineutrophil cytoplasmic antibodies(ANCA).^1^ It presents as a multisystem disorder resulting in significant morbidity and mortality.^1^ We describe herein a case of GPA presenting with hemoptysis and severe renal insufficiency that showed remarkable improvement with timely management.

## CASE REPORT

A 40 year-old female referred from a local hospital, presented in emergency with complaints of shortness of breath at rest and haemoptysis for the previous five days. The patient was in her usual state of health twenty days ago when she developed generalized swelling of body, headache and subconjunctival hemorrhage. She was treated for new onset hypertension and ten days later, after an episode of hemoptysis, presented to a local hospital. She received antibiotics for pneumonia and then referred to our hospital due to worsening hypoxia and acute kidney injury (AKI).

On presentation she was an obese lady with a BMI of 34 and marked dyspnea. On examination, she was conscious, blood pressure of 130/80mmHg and regular pulse of 120beats/minutes. She was afebrile, respiratory rate of 36/minutes and finger pulse oximetry saturation of 74% on room air. She also had pallor, periorbital puffiness and lower limb dependent edema. Chest examination revealed bi basilar end inspiratory crepitations. Her anti hypertensive medications included Amlodipine 5mg daily.

**Fig.1:**
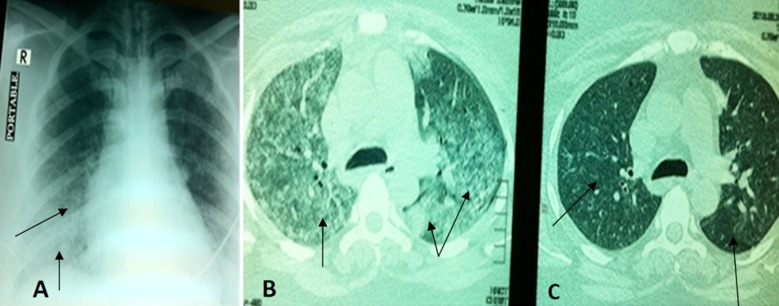
A) Chest x-ray revealed lower zones infiltrates more on right side (shown by arrows). (B) Patchy shadowing in lower zones (as shown by arrows) on high resolution computer tomography (HRCT), indicating alveolar hemorrhage. (C) Remarkable improvement noted on HRCT, showing disappearance of Hemorrhage as shown by arrows as compared to Fig.1-B.

Initial investigations were as follows: haemoglobin (Hb) 11.2g/dL; white blood cells (WBC) 24×10^3^/dL Neutrophils 86%; serum creatinine 6.7mg/dL; blood urea 210mg/dL; serum sodium 130mmol/L; serum potassium 3.7meq/L. Urine examination showed presence of many RBCs/HPF and proteins +3 and RBC casts. Chest x ray revealed bilateral middle and lower zone infiltrates (Fig.1-A) Initial differential diagnosis was acute glomerulonephritis either post infectious related to pneumonia versus rapidly progressive glomerulonephritis (RPGN) due to vasculitis. Broad spectrum antibiotics were initiated and autoimmune serology were sent. Sputum for gram staining and cultures were also sent. Arterial blood gas was suggestive of combined metabolic acidosis (high anion gap) and respiratory alkalosis. Ultrasonographic scan of the abdomen revealed normal sized kidneys with grade two renal parenchymal changes.

High resolution computed tomography (HRCT) chest done on the day of admission revealed patchy ground glass haziness in a geographic distribution confirming crazy paving, most likely alveolar hemorrhage (Fig.1-B). Empirically methylprednisolone 750mg/day for three days followed by oral 60 mg per day of prednisolone; and plasmapheresis was initiated on second admission day due to RPGN and Pulmonary vasculitis. HRCT chest was repeated on third admission day which revealed remarkable improvement (Fig.1-C). On fifth admission day, we received the results of ANCA titers which came out to be very high,(C ANCA:265.5 IU). A third session of plasmapheresis was done after an interval of one day and cyclophosphamide given on the sixth day (Table-I). She had both clinical improvements in terms of her oxygen requirement, urine output and falling serum creatinine.

**Table-I:** Serum creatinine, Protein excretion and fluid balance during hospitalization and at follow up after 15 months.

Date	Serum Creatinine (mg/dL)	Protein to creatinine ratio	Intake (ml)	Output (ml)
16/4/13	6.7		650	800
17/4/13^+@^	5.6	5.5 grams	2250	1750
18/4/13^+^	4.4		2150	1450
19/4/13	4.6		2300	1200
20/4/13^+^	3.7		2050	1550
21/4/13	3.41		1250	4300
22/4/13^#^	3.0		1360	3200
23/4/13	2.0		970	800
25/4/13	1.7		1100	3600
26/4/13^€^				
28/4/13	1.5		1650	2200
23/9/2013	1.1	0.8 grams		
*20/10/13	1.1			
*14/7/14	1.0	0.4grams		

* Follow-up visit.

€ Renal Biopsy

@ Methylprednisolone Started

+ Plasmapheresis

# Cyclophoshamide dose.

**Fig.2:**
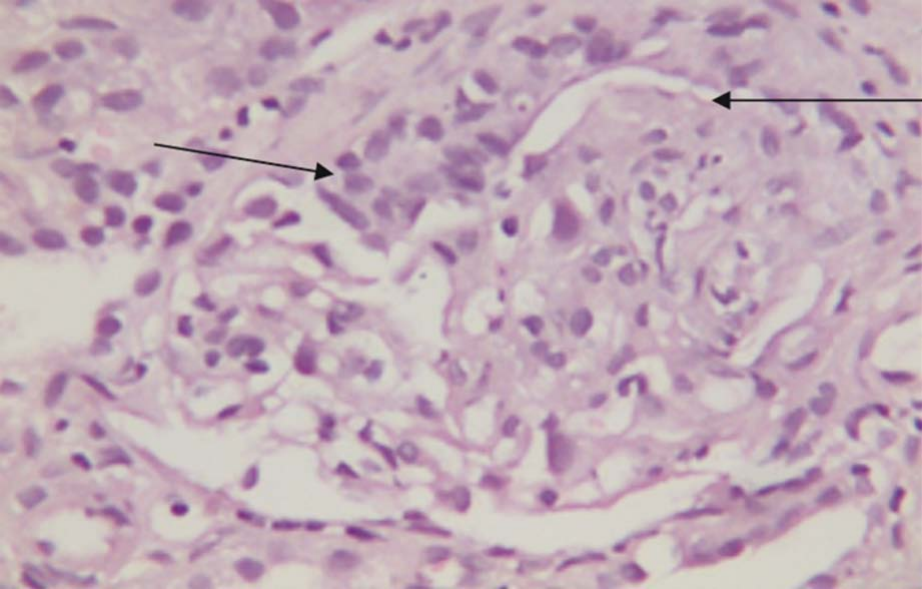
Renal biopsy slide showing Crescenteric pattern (shown by arrows).

Further plasmapheresis was not done due to the cost and rapid clinical recovery of the patient. Renal biopsy was done on eleventh admission day which revealed total of nine glomeruli, six glomeruli showed fibro-epithelial crescents with collapsed tufts. Immunofluorescence (IF) was negative confirming pauci immune crescentic RPGN (Fig.2). She was discharged on fifteenth day of admission on prednisolone 60mg/day and monthly scheduled doses of cyclophoshamide.

Follow up after six months and six doses of cyclophoshamide, revealed cushingoid face, maintained oxygen saturation on room air and serum creatinine had improved to 1.1mg/dL. Urine examination showed few RBCs and spot urine for protein to creatinine ratio 0.8 gram. At twelve months follow up and a total 8 doses of cyclophosphamide monthly, currently, she was stable on Azathioprine and 5 mg of prednisolone, follow up ANCA were in normal range. She however presented at fourteenth months with severe community acquired pneumonia (CAP) and rapidly deteriorated requiring mechanical ventilation and died.

## DISCUSSION

GPA is classified as small vessel vasculitis preferentially involving capillaries, arterioles and venules. Clinically it can involve multiple systems, commonly pulmonary in 78%, upper respiratory tract in 86% and kidney in 61% of cases.^1^ Most of the patients (90%) seek medical attention for nasal and sinus symptoms with or without involvement of lower respiratory tract.^2^ Lower respiratory tract presents as hemorrhagic infiltrates and cavitating lesions leading to haemoptysis and respiratory failure in 45% of cases.^1^ Renal manifestations are haematuria, proteinuria and RPGN in 50% of cases while 25% of cases may reach end stage renal disease (ESRD) in 3-4 years.^3^ Our patient presented predominantly with pulmonary renal syndrome as lower respiratory tract involvement and RPGN. Other systemic presentation of GPA as Cardiovascular system (CVS), Gastrointestinal (G.I), Central Nervous system (CNS) is as prescribed in a recent review by Langford C.^1^

The mainstay of GPA diagnosis is serologic evaluation. Definitive diagnosis is made on a cytoplasmic pattern of two antineutrophil cytoplasmic antibodies (ANCA). ProteinS3 ANCA, which is reactive toward proteinase-3 (PR3) or myeloperoxidase (MPO), called as c ANCA and p ANCA respectively.^4^ C ANCA has sensitivity of 91% and specificity of 99% for GPA, still p ANCA is positive in 25% of cases of GPA and about 5% of cases of GPA are negative for both c ANCA and p ANCA and diagnosis in these cases is made on strong clinical interpretation.^4^

Renal biopsy usually shows segmental or globular glomeuralr necrosis with crescent formation, immunofluroscent studies are negative for immune deposits and complements, and hence called as pauci immune.^1^ Papillary necrosis due to fibrinoid necrosis of vasa recta, focal segmental glomerular sclerosis (FSGS) or renal limited vasculitis may be other presentations of GPA.^5^ Lung biopsy is more invasive and usually not helpful due to patchy involvement of respiratory tract.^1^ Invariably the diagnosis is based on clinical presentation, ANCA serology and renal biopsy. In our patient, the clinical presentation and very high serology was enough to guide our management and RPGN on renal biopsy further consolidated the diagnosis.

Significant improvement in morbidity and mortality has been observed recently with the use of Immunosuppressive medications and plasmapheresis. Plasmapheresis increased the chances of renal recovery when used along with induction regimen as recently shown by Pepper RJ, et al.^6^ Patients who receive cyclophoshamide and corticosteroid for induction, the remission is seen in 75% by three months, similar to our patient and 90% by six months. Unfortunately nearly half (49%) of these patients attaining remission will have at least one relapse. In maintenance phase Azathioprine or methotrexate use is well established.^7^ Greater risk of relapse has been observed in patients with C- ANCA positive or pulmonary involvement. Increasing interest is developing in early detection of relapse for timely management with the help of biomarkers such as CXCL13 (BCA-1), matrix metalloproteinase-3 (MMP-3) and tissue inhibitor of metalloproteinases-1(TIMP-1).^8^ Increasing level of ANCA titers may also help in predicting the early relapse though may not be sensitive^4^

Rituximab (monoclonal antibody CD20) is a new addition in the armamentarium of immunosuppressive medications for GPA.^5^ Recent trials have shown its efficacy as induction therapy alone and also in patients with relapse.^7^,^9^ Due to the high cost of rituximab, cyclophoshamide still preferred as induction therapy, where as in resistant cases Rituximab is the drug of choice. Anti TNF alpha biologics (Infliximab and etanercept) and mycophenolate mofetil have also been used in resistant cases.^9^

A search through PubMed and Pak Medinet revealed four articles of ANCA associated vasculitis from Pakistan; by two centers, representing only twenty five patients.^2^,^3^,^10^ This does not mean that prevalence of ANCA associated vasculitis is much less, we and our other colleagues have managed several cases of ANCA associated vasculitis,(verbal communication). It is necessary that one of the tertiary care centre from Pakistan take the responsibility of maintaining a registry of all the ANCA associated vasculitis cases to document its clinical presentation and response to current treatment regimen in our population.

In conclusion, early detection of GPA is essential since it may rapidly deteriorate as in our patient. High level of index of suspicion and early initiation of management significantly improves morbidity and mortality. Plasmapheresis should be considered early on if AKI due to vasculitis is diagnosed.
